# Social Cognition in Neurodevelopmental Disorders and Epilepsy

**DOI:** 10.3389/fneur.2021.658823

**Published:** 2021-04-14

**Authors:** Grazia Maria Giovanna Pastorino, Francesca Felicia Operto, Chiara Padovano, Valentina Vivenzio, Chiara Scuoppo, Nazareno Pastorino, Michele Roccella, Luigi Vetri, Marco Carotenuto, Giangennaro Coppola

**Affiliations:** ^1^Child and Adolescent Neuropsychiatry Unit, Department of Medicine, Surgery and Dentistry, University of Salerno, Salerno, Italy; ^2^Department of Cultural Heritage Sciences, University of Salerno, Salerno, Italy; ^3^Department of Psychological, Pedagogical and Educational Sciences, University of Palermo, Palermo, Italy; ^4^Department of Mental Health, Physical and Preventive Medicine, Clinic of Child and Adolescent Neuropsychiatry, Università degli Studi della Campania “Luigi Vanvitelli”, Naples, Italy

**Keywords:** social cognition, epilepsy, autism spectrum disorder, specific learning disorder, children

## Abstract

**Introduction:** The purpose of our study was to perform a comparative analysis of social cognition in children and adolescents with epilepsy, autism spectrum disorder (ASD), specific learning disorder (SLD) and in typical development (TD) controls. The secondary aim was to relate social cognition to some clinical and demographic characteristics.

**Methods:** Our work is a transversal observational study. The recruits were 179 children and adolescents aged between 6 and 18 years diagnosed with epilepsy, ASD, or SLD and 32 subjects with TD. All the participants underwent neuropsychological assessment of Emotion Recognition (ER) and Theory of Mind (ToM) skills.

**Results:** All three clinical groups performed significantly worse than controls in ER and ToM. The ASD group achieved significantly lower performance than the other groups; however, the scores of SLD and epilepsy groups were comparable. The ER performances are related to non-verbal intelligence only in the group with epilepsy.

**Conclusion:** Children and adolescents with focal epilepsy, SLD, or ASD may present a deficit of varying extent in emotion recognition and ToM, compared with TD peers. These difficulties are more pronounced in individuals with ASD, but impairment worthy of clinical attention also emerges in individuals with SLD and epilepsy.

## Introduction

The term Social Cognition (SC) includes a set of cognitive skills and competences necessary to recognize and use socially relevant information to respond appropriately in social situations (1); it includes a wide range of interrelated functions, that comprise a basic emotion perception (e.g., gaze processing, face processing, affect recognition, visual fixation to social stimuli, and detection of biological motion) and more complex social cognitive processes [e.g., social orientation, complex social judgments, perception of social cues, attributional style, and attribution of mental states or Theory of Mind (ToM)]. Although ToM is generally considered to be an independent cognitive domain, some general cognitive skills are required for good performance on social cognition tasks, such as vigilance, attention to the other, abstract reasoning, working memory, and executive inhibition (2–6); however, the relationship between social and non-social cognition remains complex and still unclear.

The recognition of emotions allows us to identify other's emotions, moods, and states of mind through facial expression (7). Some neural structures, such as the amygdala, the insular cortex, and the basal ganglia, have been identified in the process of recognizing emotions (8). This ability develops from childhood to adolescence: toddlers are more easily able to recognize happiness, and as they grow up, they become more capable of identifying other emotions: sadness, fear, disgust in both boys and girls (9).

The term “Theory of Mind” means the ability to correctly understand and attribute mental states, intentions and desires to others (10, 11). Basic skills of ToM seem to develop from the age of three and continue to be refined until late adolescence/young adulthood (12). A set of brain regions appear to be involved in ToM tasks, including the bilateral temporo-parietal junction, posterior cingulate cortex and medial prefrontal cortex (13, 14).

Several recent studies investigated SC skills in children and adolescents with neuropsychiatric disorders.

Much scientific evidence has confirmed that SC deficits are a distinctive feature of subjects with autism spectrum disorders (ASD). These subjects, in fact, characteristically experience difficulties in the most basic SC skills, which are independent of other cognitive abilities and of general intelligence. In particular, children and adolescents with level 1 ASD, despite having a normal intelligence quotient, may have difficulty in correctly decoding other people's facial expressions as well as in understanding their moods, feelings, intentions, and thoughts, when they are compared with their typical development (TD) peers (15).

Only in recent years have SC difficulties been identified in children with other neurodevelopment disorders, such as Attention Deficit/Hyperactivity Disorder and specific learning disorder (SLD) (16, 17), and in other neurological conditions that emerge in childhood, such as epilepsy (18, 19).

Emotion recognition and Theory of Mind can be compromised in people with epilepsy, and it is hypothesized that an epileptiform activity in early childhood may influence the plasticity and maturation of social cognition neural networks (20–22). Neuroimaging studies suggested that the right medial temporal lobe is mainly involved in the emotions of fear, and lesions can interfere with the entire neural network of SC (23). Moreover, lesions of the amygdala altered the activation of regions engaged in SC abilities (24).

Our comparative study evaluated social cognition skills (Emotion Recognition and Theory of Mind) among children and adolescents with level 1 ASD, SLD, epilepsy and TD controls. The secondary aim of this study was to explore the relationship between social cognition performances and other factors such as age, sex, non-verbal intelligence, epilepsy-related factors, and SLD characteristics.

## Methods and Materials

### Study Design

This cross-sectional observational study aimed to exploring social cognition skills (recognition of emotions through facial expressions and Theory of Mind) in children and adolescents with focal epilepsy, specific learning disorder, and level 1 autism spectrum disorder compared to typically developing controls.

### Participants

We recruited 240 children and adolescents aged 6–18 years, with a diagnosis of focal epilepsy (*n* = 62), SLD (*n* = 63), Level 1 ASD (*n* = 54) and 61 TD controls, homogeneous by sex, age, and socioeconomic status (Table 1). Patients were enrolled at the Child and Adolescents Neuropsychiatry Unit of the University of Salerno from December 2017 to September 2020. The diagnosis of focal epilepsy was based on seizure semiology and recurrence and EEG features. Specific learning disorders were diagnosed following the intellectual profile (WISC-IV) and the assessment of reading-writing and calculation skills (MT reading clinical tests, DDE-2, Battery for the assessment of writing and spelling skills-2, AC-MT). The diagnosis of ASD was based on the DSM-5 (25) criteria and supported by clinical observations and by standardized tests (ADOS-2, ADIR-R, WISC-IV). Patients with dual diagnoses (e.g., autism spectrum disorder and epilepsy, specific learning disorder, and epilepsy) were excluded.

**Table 1:** Sample characteristics.

	**Epilepsy**	**SLD**	**Autism**	**Control**	**Statistics**
Sample size	62	63	54	61	
Males	40 (65%)	34 (54%)	38 (70%)	32 (52%)	*p =* 0.127 *χ =* 5.700
Females	22 (35%)	29 (46%)	16 (30%)	30 (48%)	
Age in years (M ± SD)	12.74 ± 3.4	11.51 ± 2.83	11.70 ± 5.05	11.56 ± 3.28	*p =* 0.130 *χ =* 5.652
Years of schooling	9.45 ± 3.16	8.51 ± 2.83	8.54 ± 4.64	8.38 ± 3.12	*p =* 0.208 *χ =* 4.546
Maternal education level^*^	14.11 ± 3.36	13.84 ± 3.43	14.46 ± 2.91	13.54 ± 2.55	*p =* 0.706 *χ =* 4.062

*SLD, specific learning disorder; M, mean; SD, Standard Deviation*;

* *Calculated as years of maternal education*.

The control group was recruited among healthy subjects who came to our clinic for a screening project on learning difficulties, in which epileptic disorders, ASD, and SLD were excluded.

The exclusion criteria for all four groups were the following: presence of additional conditions of neurological or psychiatric interest (cerebral palsy, neurodegenerative diseases, migraine, intellectual disability, attention deficit/hyperactivity disorder, anxiety, depression, and psychosis) or other relevant medical conditions (endocrinopathies, metabolic, hepatic, cardiac, or renal diseases). Other variables, such as age, sex, school years, intellectual level, and level of maternal education were considered in the four groups.

The parents of all the participants provided their written informed consent after receiving a full description about the purpose and the protocol of the study. The study design was approved by the Campania Sud Ethics Committee, and it was conducted according to the rules of good clinical practice, in line with the Declaration of Helsinki. Sample characteristics are summarized in Tables 1, 2.

**Table 2:** Epilepsy and specific learning disorder characteristics.

**Epilepsy**		**Subtype SLD**	
Age of onset (M ± SD)	6.74 ± 3.96		
Long Duration (M ± SD)	6.00 ± 4.04	Mixed	37 (59%)
**Involved hemisphere**			
Left	30 (48%)	Dyslexia + Dysorthography	17 (27%)
Right	32 (52%)		
**Involved lobe**			
Temporal	38 (61%)	Dyslexia	9 (14%)
Frontal	15 (24%)		
Occipital	9 (15%)		
**Crisis frequency (M** **±** **SD)**			
Monthly	20 (32%)		
Weekly	26 (42%)		
Daily	16 (26%)		
**Pharmacological therapy**			
Monotherapy	36 (58%)		
Polytherapy	26 (42%)		
ASMs (M ± SD)	1,6 ± 0.78		
NMR positive	16 (26%)		
Frontal lobe	3 dysplasia cortical		
Temporal lobe	5 temporal sclerosis		
	2 cortical dysplasia		
	6 hypoxic-ischemic damage		

*SLD, specific learning disorder; s, sample size; M, mean; SD, standard deviation; ASM, anti-seizure medication; NMR, nuclear magnetic resonance*.

### Neuropsychological Assessments

The Second Edition of the Developmental NEuroPSYchological Assessment battery (Nepsy-II) (26) was employed for social cognition assessment. This battery analyzes several domains: social cognition skills are evaluated through a subtest of discrimination; recognition and contextualization of emotions through facial expressions (Emotion Recognition: ER) and a subtest that evaluates the ability to understand mental functions such as beliefs, intentions, deceptions, and emotions (Theory of Mind: ToM). The results of both tests are expressed as raw scores and then converted to age-weighted scores. Weighted scores are expressed on a numerical scale, with mean = 10 and standard deviation (SD) = 3. Age-standardized scores are classified as normal range: ≥8; between 7 and 4 at the lower limits of the norm (−1 standard deviation); <4 below normal (−2 standard deviations).

Progressive Raven Standard Matrices (SPM) (27) were administered to all participants to assess non-verbal cognitive abilities: this test includes five series of 12 elements each, which require an increasing cognitive ability to code and analyze information. Raw scores were converted into percentiles (pc) and age-weighted scores (pp) with mean = 100 and standard deviation = 15.

### Statistical Analysis

All neuropsychological data were expressed as mean and standard deviation. The percentage of subjects who scored below normal (<2 standard deviations) was considered. To verify the data distribution, the Kolmogorov-Smirnov normality test was preliminarily performed. Since data were not normally distributed, non-parametric methods were employed for statistical analysis. We performed chi-square tests for the comparison between proportions, and the Kruskal-Wallis test for independent samples was used to compare the mean scores of several independent groups. *Post-hoc* analysis was performed using the Mann-Whitney U test. The two-tailed Spearman rank correlation test was used to evaluate the relationship between different variables. The correlations were interpreted according as follows: *r* < 0.2, low; 0.21–0.40, fair; 0.41–0.60, moderate; 0.61–0.80, good; 0.81–1.00, very good. All data were analyzed using the Statistical Package for Social Science software, version 23.0 (IBM Corp.); *p*-values ≤ 0.05 were considered statistically significant.

## Results

Clinical and Demographic Characteristics of Our Sample are Synthesized in Tables 1, 2.

### Comparison vs. Control Group

**Figure 1:**
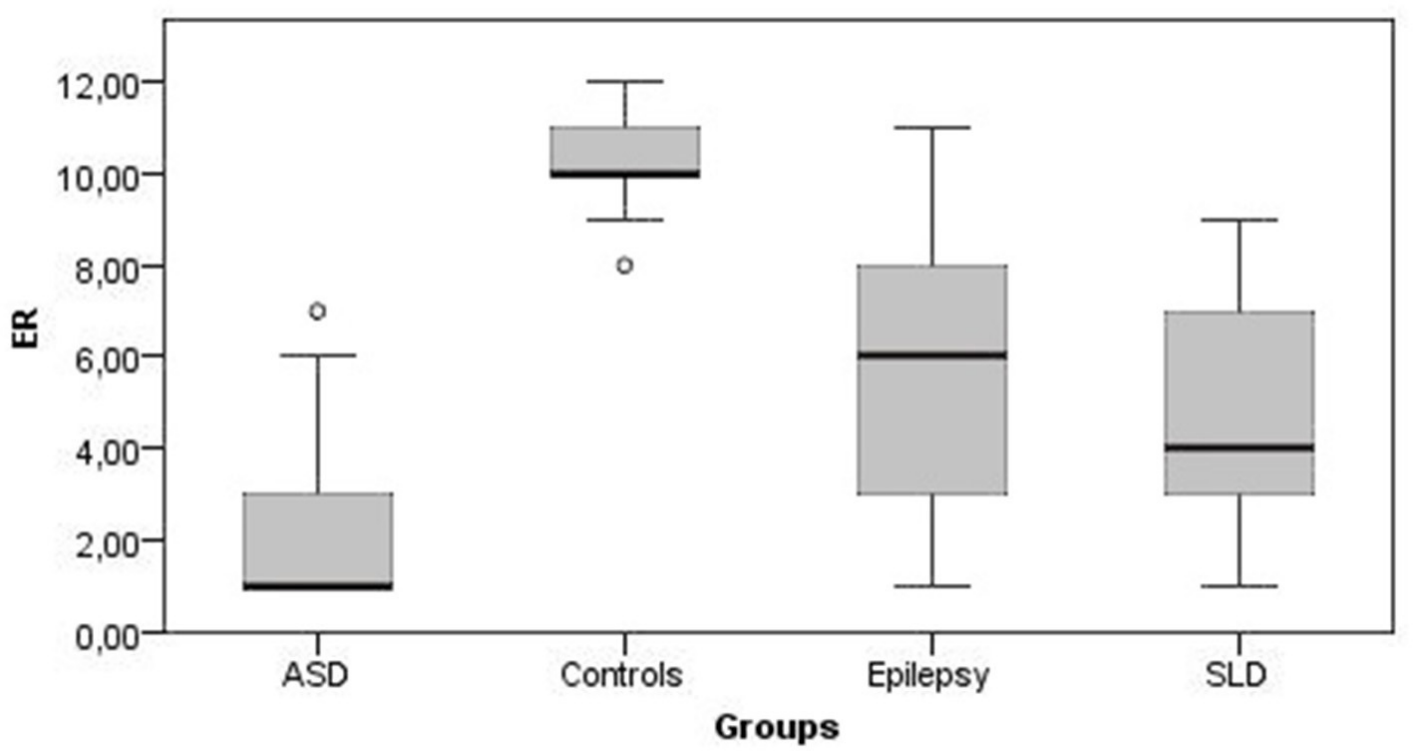
Emotion recognition mean scores. ER, emotion recognition; ASD, autism spectrum disorder; SLD, specific learning disorder.

**Figure 2:**
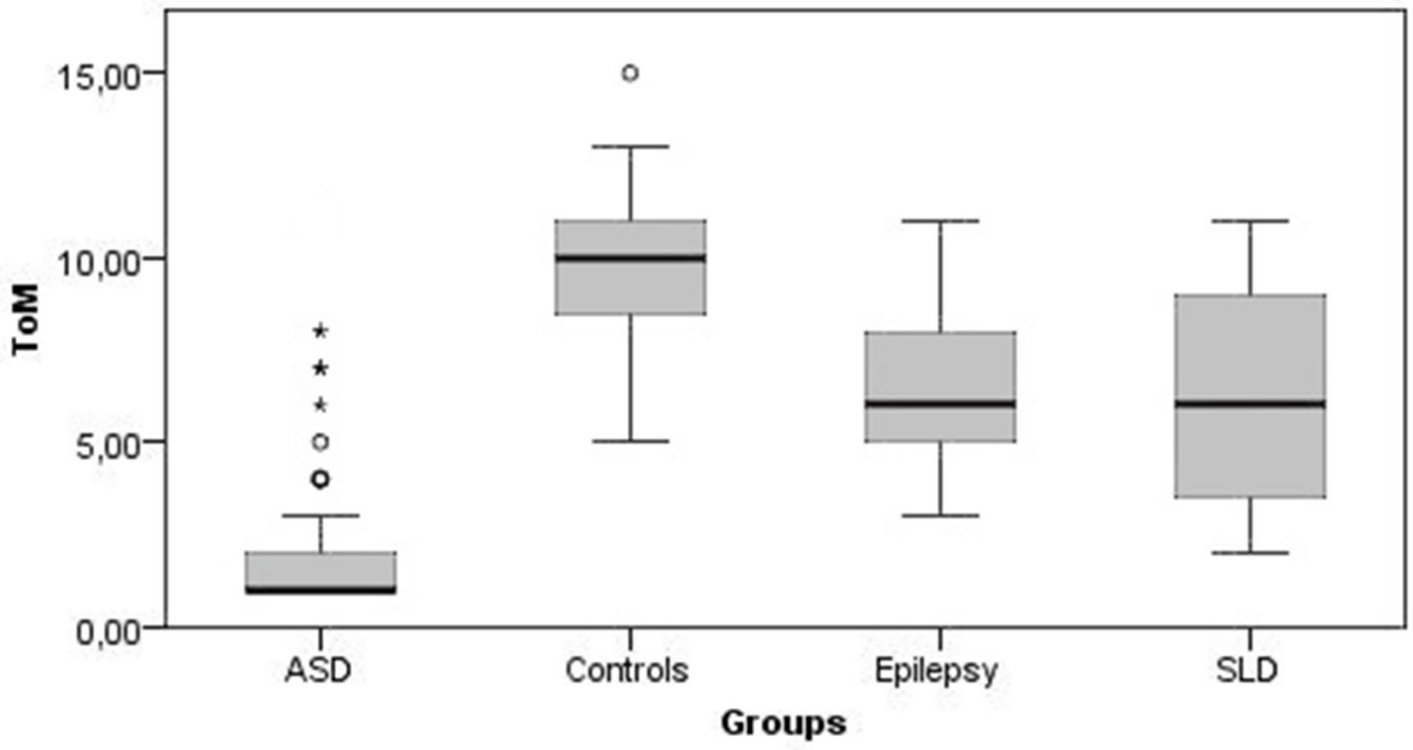
Theory of Mind mean scores. ToM, theory of mind; ASD, autism spectrum disorder; SLD, specific learning disorder.

Figures 1, 2 show all the average scores obtained on the NEPSY-II neuropsychological test (ER and ToM) among the group with epilepsy, ASD, SLD, and TD.

In ER skills, 42% of children with epilepsy, 38% of children with SLD, and 76% of children with ASD obtained a score below the norm (<2 standard deviations); in ToM skills the percentage of subjects that performed under the norm were 9, 25, and 82% for epilepsy, SLD, and ASD groups, respectively.

The comparison between the average scores of the epilepsy group and the TD group shows that the mean total score on the ER subtest was at the lower limits of the norm for the epilepsy group (mean score = 5.35 ± 2.68; < 1 standard deviation) while it was within the norm for the TD group (mean score = 10.36 ± 1.08), and this difference was statistically significant [U(123) = 126, *p* < 0.001]. The mean total scores on the ToM subtest were at the lower limits of the norm for the epilepsy group (mean score = 6.39 ± 2.18; < 1 standard deviation) while the mean score for the control group was in the range of norm (mean score = 9.67 ± 1.74), and this difference was statistically significant [U(123) = 482, *p* < 0.001]. On the contrary, non-verbal intelligence was preserved in both groups, with mean scores at SPM test falling within normal range, with no statistically significant differences between the two groups [U(123) = 1,744, *p* = 0.456].

Also, in the SLD group the average total scores of the ER was at the lower limits of norm (mean score = 4.59 ± 2.52; <1 standard deviation) and was significantly lower than TD controls [U(124) = 26, *p* < 0.001). The average total scores of the ToM subtest were at lower limits of norm for the group with SLD (mean score = 6.21 ± 2.87; <1 standard deviation) and was significantly lower compared with the control group [U(124) = 666.5, *p* < 0.001]. Conversely, non-verbal intelligence was within the norm in both groups with no statistically significant differences [U(124) = 1,708.5, *p* = 0.286].

The average total scores of the ER subtest were placed below the norm for the group with ASD (mean score = 2.46 ± 2.38; <2 standard deviations) and was significantly lower than controls [U(115) = 86, *p* < 0.001]. The average total scores of the ToM subtest were below the norm for the group with ASD (mean score = 2.33 ± 2.43; <2 standard deviations) and was significantly lower than in the control group [U(115) = 117.5, *p* < 0.001]. Although non-verbal intelligence was preserved in both groups, subjects with AS scored significantly higher average SPM test scores than controls [U(115) = 600, *p* < 0.001].

### Comparative Analysis

The results of comparative analysis showed statistically significant differences among the three groups analyzed (Epilepsy, SLD, and ASD) in all the parameters considered (ER, ToM, non-verbal intelligence) as summarized in Table 3.

**Table 3:** Comparison among groups with nonparametric analysis of variance tests.

	**Epilepsy**	**SLD**	**ASD**	**Statistics**
	**M ± SD**	**M ± SD**	**M ± SD**	**Kruskal–Wallis H**
ER	5.35 ± 2.68	4.59 ± 2.52	2.46 ± 2.38	**χ^2^ = 10.79, *p* < 0.001**
Neutral	1.77 ± 1.46	1.96 ± 1.38	2.31 ± 1.40	**χ^2^ = 25.99, *p* < 0.001**
Happiness	0.45 ± 0.72	0.72 ± 0.80	1.54 ± 0.88	**χ^2^ = 63.75, *p* < 0.001**
Sadness	3.29 ± 1.15	3.72 ± 1.36	2.57 ± 1.31	**χ^2^ = 37.32, *p* < 0.001**
Fear	1.65 ± 1.07	2.19 ± 1.38	2.43 ± 1.25	**χ^2^ = 54.73, *p* < 0.001**
Anger	2.29 ± 1.45	2.70 ± 1.37	2.83 ± 1.40	**χ^2^ = 34.39, *p* < 0.001**
Disgust	2.03 ± 0.97	2.36 ± 1.45	2.85 ± 1.45	**χ^2^ = 25.10, *p* < 0.001**
ToM	6.39 ± 2.18	6.21 ± 2.87	2.33 ± 2.43	**χ^2^ = 103.00, *p* < 0.001**
SPM	90.48 ± 8.65	93.95 ± 6.96	107.85 ± 16.38	**χ^2^ = 53.03, *p* < 0.001**

*ER, emotion recognition; ToM, theory of mind; SPM, standard progressive matrices. Significant p-values are in boldface*.

A *post-hoc* analysis revealed that in the ER and ToM subtests, the epilepsy and SLD groups obtained scores that did not differ significantly from each other but were significantly higher than those obtained by the ASD group (Table 4). On the contrary, in the SPM test for measuring non-verbal intelligence, the scores obtained by the epilepsy and SLD groups did not differ significantly from each other but were significantly lower than those of the group with ASD (Table 4). With respect to single emotions the epilepsy group scored significantly better (fewer errors) than SLD, which, in turn, scored better than the ASD group in identifying happiness (epilepsy > SLD > ASD); in identifying sadness expressions, the epilepsy group and the SLD group obtained significantly better scores than the ASD group (SLD = epilepsy > ASD); as for the identification of fear, anger, and disgust, the group with epilepsy obtained better scores than the groups with ASD and SLD, which performed similarly.

**Table 4:** *Post-hoc* analysis between groups (Mann-Whitney U test).

**Emotion recognition (ER)**			**Theory of mind (ToM)**			**Nonverbal intelligence (SPM)**		
1 vs. 2	U ***=*** 1.642	*p =* 0.121	1 vs. 2	U ***=*** 1.847	*p =* 0.598	1 vs. 2	U ***=*** 1.586	*p* = 0.069
**1 vs. 3**	**U = 524**	***p*** **< 0.001**	**1 vs. 3**	**U = 328**	***p*** **< 0.001**	**1 vs. 3**	**U = 5.24**	***p*** **< 0.001**
**2 vs. 3**	**U = 638**	***p*** **< 0.001**	**2 vs. 3**	**U = 399**	***p*** **< 0.001**	**2 vs. 3**	**U = 638**	***p*** **< 0.001**
**Neutral**	**Happiness**	**Sadness**						
1 vs. 2	U ***=*** 1.781	*p* = 0.380	**1 vs. 2**	**U = 1.526**	***p*** **= 0.018**	1 vs. 2	U ***=*** 1.726	*p* = 0.247
**1 vs. 3**	**U = 1.260**	***p*** **= 0.018**	**1 vs. 3**	**U = 590**	***p*** **< 0.001**	**1 vs. 3**	**U = 1.123**	***p*** **= 0.002**
2 vs. 3	U ***=*** 1.416	*p*- 0.111	**2 vs. 3**	**U = 935**	***p*** **< 0.001**	**2 vs. 3**	**U = 1.081**	***p*** **= 0.001**
**Fear**	**Anger**	**Disgust**						
**1 vs. 2**	**U = 1.504**	***p =*** **0.021**	**1 vs. 2**	**U = 1.528**	***p =*** **0.032**	**1 vs. 2**	**U = 1.336**	***p =*** **0.002**
**1 vs. 3**	**U = 1.084**	***p =*** **0.001**	**1 vs. 3**	**U = 1.321**	***p =*** **0.046**	**1 vs. 3**	**U = 1.099**	***p =*** **0.001**
2 vs. 3	U ***=*** 1.497.5	*p =* 0.253	2 vs. 3	U ***=*** 1.695.5	*p =* 0.975	2 vs. 3	U ***=*** 1.698.5	*p =* 0.989

*1, epilepsy group; 2, specific learning disorder group; 3, autism spectrum disorder group. Significant p-values are in boldface*.

### Effect of Other Variables on Social Cognition

In the epilepsy group, there was a statistically significant positive correlation between the ER score and the SPM score (Spearman's ρ = 0.568; *p* < 0.001) as well as a positive correlation between ER score and age of onset of epilepsy (Spearman's ρ = 0.761; *p* < 0.001); the ToM score was negatively correlated with the seizure frequency, reaching statistical significance (Spearman's ρ = −0.521; *p* < 0.001). Correlation analysis also revealed a positive correlation between SPM score and age of onset of seizures (Spearman's ρ = 0.446; *p* < 0.001) and negative correlation between SPM score and duration of epilepsy (Spearman's ρ = −0.365; *p* = 0.005). For the SLD group the correlation analysis showed a significant positive correlation between the ER and ToM scores (Spearman's ρ = 0.475; *p* < 0.001). For the ASD group the correlation analysis showed a significant positive correlation between the ER and ToM scores (Spearman's ρ = 0.514; *p* < 0.001). Finally, the other parameters analyzed, such as age, sex, lobe, side of origin of the epilepsy, and type of SLD, did not have a significant relationship with the performance of social cognition analyzed (*p* > 0.05).

## Discussion

Our study explored social cognition skills (ER and ToM) in children and adolescents with epilepsy, SLD, and Level 1 ASD, using a battery of neuropsychological standardized tests. The group with epilepsy was included because epilepsy is a network disorder that compromises various cognitive domains, as it happens in neurodevelopmental disorders (19, 20).

According to previous studies and also in our sample, compared with their peers, children with epilepsy disclosed difficulties in ER and ToM (21, 22, 28). Studies in the literature underscore the importance of implementing the treatment of socio-cognitive deficits through specific interventions together with the pharmacological treatment of seizures, to maximize neurodevelopmental outcomes and complete life-long management of epilepsy (29, 30).

Furthermore, in agreement with the previous data from the literature (19), there was a relation between worse ToM performances and epilepsy severity, combined with a higher seizure frequency. This relationship could be linked both to frequent epileptic discharges affecting the neural network implicated in social cognition and to the social stigma limiting interaction with peers and opportunities for social experiences (31).

In agreement with previous studies (26, 32) there was a significant relationship between worse ER abilities and a lower age of seizure onset and a longer duration of epilepsy.

In the literature there are some studies (33, 34) that showed a correlation between social cognition skills and temporal lobe regions, so in our study it was hypothesized that epilepsy of the temporal lobe could be more associated with a deficit in social cognition. Although our results do not show a relationship between ER and ToM and the focus site on epilepsy, it should be considered that there are conflicting data in the literature. Indeed, although in some research, patients with temporal lobe epilepsy have impaired social cognition, other studies have not shown an association between performance in social cognition and the source lobe of epilepsy (18).

Concerning SLD subjects there are few studies examining social cognition; Sahin et al. (16) showed that children with neurodevelopmental disorders, including SLD, had ToM deficits regardless of intelligence and language development. Bloom and Heath (35) analyzed the recognition and the understanding of facial expressions, comparing the general learning disorder group and the non-verbal specific learning disorder group with a control group. Results emerged from the study cited showed that subjects with general learning disorder performed worse in facial expressions recognition than those with non-verbal specific learning disorder group and the control group, which did not significantly differ from each other. According to data from the literature and our study also, children and adolescents with SLD showed a significant impairment in ER and ToM skills compared to the TD group, independent of SLD subtype.

Regarding the patients with level 1 ASD, several studies proved a significant impairment in social cognition abilities (36–38). Our study confirmed lower performances in ER and ToM compared with the TD group (76% of children and adolescents with ASD scored under 2 standard deviations in ER and 82% in ToM). Specifically, our sample showed poor performances in the recognition of all main emotions and neutral expressions.

From our comparative analysis it emerged that the ASD group achieved significantly lower performances (mean scores < 2 standard deviations) compared with children with SLD and epilepsy in both ER and ToM (mean scores <1 standard deviation for both). Moreover, the overall performance did not significantly differ between epilepsy group and SLD groups in ER and ToM. Furthermore, the percentages of subjects scoring below the norm were very similar between the epilepsy and SLD groups, for both ER (42 vs. 38%, respectively) and ToM (9 vs. 25%, respectively) but were higher in the ASD group (76% for ER and 82% for ToM).

To our knowledge, there were no studies that comparatively evaluate social cognition in children with epilepsy, SLD, and ASD children.

Only the study by Sahin et al. (16) compared ToM skills in children with ASD and with SLD, highlighting that the ASD group scored lower than the SLD group, although this difference was not significant.

As for the recognition of individual emotions, it seems that subjects with ASD present greater difficulties than the other two groups in recognizing neutral expressions and happiness; subjects with epilepsy and SLD show greater difficulty in recognizing sadness than subjects with ASD; finally, subjects with ASD and SLD have greater difficulties in discriminating the expressions of fear, anger, and disgust than subjects with epilepsy.

This finding suggests that individuals with ASD have generalized difficulties in recognizing all facial expressions, even those that express positive emotions, which are more easily identified. The other two groups, on the contrary, have greater difficulties in recognizing negative emotions (whose discrimination matures later) with worse performance in the group with SLD than in the group with epilepsy (17, 39).

Regarding non-verbal intelligence, all three groups performed in the normal range; in particular, the SLD group and the epilepsy group obtained similar results, while the ASD group achieved significantly higher performance. Our results highlighted a significant correlation between non-verbal intelligence and ER in children with epilepsy: the patients with greater impairment of non-verbal intelligence are those who have greater difficulties in recognizing facial expressions. Unlike the epilepsy group, the ability of social cognition in ASD and SLD subjects is not significantly correlated with non-verbal intelligence. Indeed, the ASD subjects, despite having obtained the highest scores in non-verbal intelligence compared with the other two groups, obtained the worst performance in the ER and ToM.

This finding is in line with previous studies, confirming that there was no correlation between non-verbal intelligence and SC skills in ASD individuals (40); conversely, there may be a correlation between intellectual abilities and SC in other neuropsychiatric conditions and in epilepsy (41, 42).

Overall, our results reconfirmed that the social cognition deficit is one of the peculiar characteristics of ASD children, and it can be hypothesized that it affects social communication difficulties and social interaction typical of these subjects (15).

The results of our study also suggest that difficulties in social cognition are present not only in autism spectrum disorders, but also in other neuropsychiatric disorders impairing brain development in the early stages of life, with a different severity gradient (16, 17, 42).

The strength of our study is the recruitment of a control group and the use of standardized direct neuropsychological tests.

The principal limitations of our research were the modest sample size and the cross-sectional design, which does not provide information about the evolutionary trajectories of social cognition over time. In this regard, we plan to conduct a prospective longitudinal study to follow the changes in social cognition over time, both in the three study groups and in the control group.

Another limitation of our study is that we only considered one type of test for assessing social cognition skills. In future studies more environmental tests could be used for the evaluation of RE and ToM, such as the TOMI (43); furthermore, other aspects of social cognition, such as social decision making and moral judgment, could be evaluated.

Other important future research could evaluate the influence of more variables on social cognition skills, such as some components of both receptive and expressive language (e.g., vocabulary) through standardized tests.

Finally, functional neuroimaging studies would be useful to investigate the regions and neural networks involved in the pathogenesis of ASD, SLD, and epilepsy and their relationships with social cognition, in order to allow new advances in knowledge but also to direct research toward new molecular treatments, based on functional abnormalities.

Our study has important practical implications, suggesting that early monitoring of social cognition skills in children with ASD, SLD, and epilepsy would be useful in order to undertake early intervention.

In particular, with regard to autism spectrum disorders, we found that the ToM and ER deficits are especially present in this population despite the higher IQ; in children with ASD, therefore, specific and differentiated support should be provided to assist the learning of these social skills.

## Conclusions

The results of this study suggest that children and adolescents with focal epilepsy or SLD or ASD have deficits in recognizing emotions through facial expressions and deficits in ToM, compared with TD peers. These deficits are more pronounced in individuals with ASD, but deficits worthy of clinical attention also emerge in individuals with SLD and epilepsy. The ER and ToM are fundamental aspects of social cognition and important for social relationships, which is the reason these skills must be monitored at a developmental age to provide early interventions and to guarantee children and adolescents a good quality of life.

## Data Availability Statement

The raw data supporting the conclusions of this article will be made available by the authors, without undue reservation.

## Ethics Statement

The studies involving human participants were reviewed and approved by Campania Sud Ethics Committee. Written informed consent to participate in this study was provided by the participants' legal guardian/next of kin.

## Author Contributions

FO conceptualized the work. GP analyzed the data and drafted the manuscript. CP, VV, and CS performed psychometric measurements and analyzed the data. NP drafted the manuscript and revised the language. LV researched the data in the literature. MR, MC, and GC were involved in planning and supervised the work. All authors have agreed to this final version, and participated in a meaningful way in the preparation of the manuscript.

## Conflict of Interest

The authors declare that the research was conducted in the absence of any commercial or financial relationships that could be construed as a potential conflict of interest.
